# Silence is not golden: the hissing calls of tits affect the behaviour of a nest predator

**DOI:** 10.1007/s00265-017-2313-5

**Published:** 2017-04-07

**Authors:** Karol Zub, Dorota Czeszczewik, Ireneusz Ruczyński, Anna Kapusta, Wiesław Walankiewicz

**Affiliations:** 1 Mammal Research Institute, Polish Academy of Sciences, Waszkiewicza 1, 17-230 Białowieża, Poland; 2 Faculty of Natural Science, Department of Zoology, Siedlce University, Prusa 12, 08-110 Siedlce, Poland

**Keywords:** Anti-predator strategy, Cavities, Threatening call, Flycatcher, *Apodemus flavicollis*

## Abstract

**Abstract:**

Nest predation is one of the most important mortality factors of birds. Field observations showed that tits (Paridae) produce hissing calls and, usually, have lower breeding losses than nesting *Ficedula* flycatchers, which do not make such calls. We hypothesise that differences in fledgling success can be directly attributed to the vocal reaction of tits. We tested experimentally whether the hissing calls can affect the behaviour of a potential predator, analysing the response of the Yellow-necked Mouse *Apodemus flavicollis* to playback of calls of three Parid species. The number of visits by mice to two types of cavities (with playback and control) was not significantly different, but the average time spent by mice in cavities with playback (3.9 s) was significantly shorter than in cavities without playback (26.3 s). This suggests that hissing behaviour of tits significantly changes the exploration activity of predators, which may ultimately increase the breeding success of this group of birds relative to the flycatchers.

**Significance statement:**

Nest predation is one of the most important mortality factors of small land birds, but some anti-predatory mechanisms are still poorly recognised. Numerous studies demonstrate that incubating tits make hissing sounds, when a predator is near, but despite almost a century of research, there is little evidence these calls indeed affect behaviour of predators. By using a simple laboratory experiment, we demonstrated that the hissing acoustic signals used by tits may change the behaviour of yellow-necked mice, which are an important predator of cavity-nesting birds in temperate forests. Intruding mice withdrew from cavities where hissing sounds were played back. Our results suggest that the hissing behaviour of tits can change the exploration activity of potential predators and may increase breeding success of this group of birds relative to the flycatchers, which stay silent when their nest is threatened.

## Introduction

In birds, nest predation has promoted the evolution of various morphological, physiological, and behavioural anti-predator adaptations (Martin 1987; Lima 2009; Parejo et al. 2013). Potential prey may be able to change the behaviour of a predator, but cavity-nesting birds generally remain hidden, relying on a small cavity entrance as passive nest protection. In some species, parental alarm calls can warn nestlings about presence of different predators and in response juveniles can modify their behaviour (Magrath et al. 2010; Suzuki 2011). Depending on the type of predator, nestlings can jump out of the cavity or crouch inside. Similarly, incubating female tits avoid the attack of snakes by leaving their nests in response to specific alarm calls given by their mates (Suzuki 2011, 2015). Such behaviour enhances survival and directly affects fitness. Some bird species (Wood Warbler *Phylloscopus sibilatrix*, Parids, Burrowing Owl *Athene cunicularia*) use acoustic signals, which may effectively change the behaviour of predators (Cox 1930; Sibley 1955; Rowe et al. 1986; Krams et al. 2014). This type of reaction in the presence of an intruder is especially prevalent in tits (Paridae). Numerous studies demonstrate that incubating tits bang their wings inside the cavity and make hissing sounds, similar to that of snake or weasel (Odum 1942; Sibley 1955; Broughton 2005, 2012). Despite almost a century of research, there is little evidence that hissing calls actually affect the behaviour of predators (Jourdain 1929; Apel and Weise 1986). Krams et al. (2014) showed that hissing Great Tit *Parus major* females were killed less often than silent females and suggested that hissing calls could deter some predator attacks, potentially increasing the survival rates of nesting birds or their offspring.

In the Białowieża Forest (E Poland), there is a large diversity of cavity-nesting birds (Czeszczewik et al. 2015) and also their nest predators. Species preying upon cavity nesters’ eggs or young include rodents (Yellow-necked Mouse *Apodemus flavicollis*, Forest Dormouse *Dryomys nitedula*, Fat Dormouse *Glis glis*, Red Squirrel *Sciurus vulgaris*), mustelids (Pine Marten *Martes martes*, Weasel *Mustela nivalis*), and the Great Spotted Woodpecker *Dendrocopos major* (Walankiewicz 2002a; Wesołowski 2002; Czeszczewik et al. 2008; Wesołowski and Rowiński 2012; Maziarz et al. 2016). Previous work has demonstrated that in our study area, nest predation in tits is usually lower than in *Ficedula* flycatchers (Walankiewicz 2002b; Wesołowski 2002; Czeszczewik 2004; Wesołowski and Rowiński 2012; Maziarz et al. 2016). In years of high rodent abundance (2008–2011), breeding losses of the Great Tit caused by nest predation ranged up to 41% (Maziarz et al. 2016), while breeding losses of the Collared Flycatcher reached 60% (WW and DC, unpubl. data). Breeding losses of the Marsh Tit and the Blue Tit were lower (up to 26% plundered broods; Wesołowski 2002; Wesołowski and Rowiński 2012). In rodent outbreak years, one of the most important predators of flycatcher broods is the Yellow-necked Mouse (Walankiewicz 2002a, b), which can climb trees and enter nest cavities (Borowski 1962; Czeszczewik et al. 2008). This rodent species can reach a density of a few hundred individuals/ha in the Białowieża National Park (Pucek et al. 1993; Walankiewicz 2002b). During breeding period, Parids and flycatchers behave quite differently—when a predator looks into a cavity, the tits make aggressive hissing displays, while flycatchers stay silent. We assumed that differences in nestling losses between tits and flycatchers could arise, at least partially, from these differing behaviours.

To determine whether the hissing calls of tits affect the behaviour of Yellow-necked Mice, we observed the behaviour of mice visiting artificial tree cavities with or without the hissing calls of tits. Some evidence already supporting this hypothesis comes from Krams et al. (2014) who found that tit hissing calls prevented the attacks of feral cats; however, they did not include a negative control treatment in their experiments.

## Material and methods

### Experimental design

**Fig. 1:**
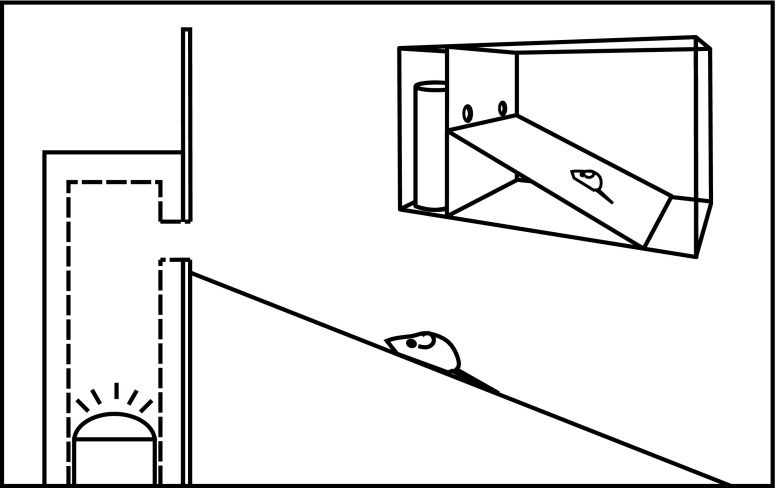
Design of the experimental chamber. General view (*top right*) and vertical cross section (*main picture*)

Our experiment mimicked a natural situation where mice explore cavities and encounter those occupied by tits or flycatchers. In the experiment, we simulated the hissing response of tits with playback of recorded hissing calls; no playback represented the response of flycatchers. We also observed directly in an arena how Yellow-necked Mice responded to a playback of the hissing call of adult tits and how they behaved when there was no call. To do this, we placed two sections of black alder trunk (0.5 m long × 0.25 m diameter) with artificial cavities (5 cm entrance diameter) in a Plexiglas chamber (2 × 1 × 1 m). These trunks were covered by a wooden wall (made of plywood) so that only the entrances were visible (Fig. 1). We also installed a 1-m long ramp, covering the entire floor and leading to both artificial cavities. After each trial, we replaced tree trunks with new ones and cleaned the arena, to avoid the possible confounding effect of olfactory cues left by the mice on the wood. Within each cavity, we installed a speaker (Maxell MXSP-101W), connected to an mp3 player (Creative ZEN Style EZ 300) and motion detector (Fig. 1).

Each time a mouse entered the cavity, the motion detector initiated the playback, which continued as long as the mouse stayed in the hole. For playbacks, we used the hissing calls of three different species: Great Tit (in 25 trials), Marsh Tit *Poecile palustris* (in 23 trials), and Blue Tit *Cyanistes caeruleus* (in 16 trials). Every hissing call of tits was replicated using three different recordings (variants). During each trial, the playback (simulating an occupied cavity) was assigned randomly to either the left or right trunk. We used 64 mice, and each animal was exposed to only one type of call in one trial. Imperfect trials due to technical problems (e.g. escape of mouse from arena during experiment, blurred video recording) were not included in the analysis and the exclusion of these trials resulted in unequal numbers of trials for each of the three species of tits.

The hissing calls used for playback were recorded in the field using a Zoom H2 Handy Recorder. The presence of the recorder close to the cavity entrance stimulated hissing by the adult tits. The recorder was placed 10 cm from the entrance, which gave a distance of 25–30 cm from a bird. Typically, we made five recordings for each of the three tit species; however, we used only the three best (i.e. highest audio quality) recordings for each species in the playback experiments. Hissing calls of tits were recorded in the Białowieża National Park (NE Poland) in spring 2012 and 2014. Calls were recorded as wav files (sampling rate 44.1 KHz) and for playback converted into mp3 format. Files converted to mp3 format retained a high fidelity in sound parameters (Gonzales and Cervera 2001).

The calls played in the laboratory were amplified to a sound level that was similar to that produced by birds in the field. Recordings of Great Tit calls were used for standardisations of playback settings. To determine the appropriate volume level for playback, the volume of the mp3 player was set to one of ten predetermined levels and calls were played from a speaker placed in the tree trunk. The playback was then recorded 10 cm from the cavity entrance by using a Zoom H2 Handy Recorder. This procedure replicated that used in the field to make recordings of tits hissing from the nest. The sound level for each playback sequence was measured from recordings made outside the cavity by using a one-dimensional transformation function (Root mean square (logarithmic), average time 125 ms) in SasLab Pro software (Avisoft Bioacoustic, Germany). The highest values of sound level recorded in the laboratory were compared with the highest values of respect call recorded in the field. The species-specific playback setting that gave the value most similar to that recorded in the field (deviation = 0.8 dB) was used in the experiments for all three bird species (Great Tits, Blue Tits, and Marsh Tits). Call duration and the time between calls were measured manually from waveforms (FFT length = 256). Peak frequency (frequency of maximum power) and maximum frequency were measured, using an automatic parameter measurement procedure (Zollinger et al. 2012) implemented in SasLab Pro. A threshold of −30 dB below the peak amplitude was used for measuring maximum frequency, to avoid problems of covariation between amplitude and maximum frequency (FFT length = 512).

**Fig. 2:**
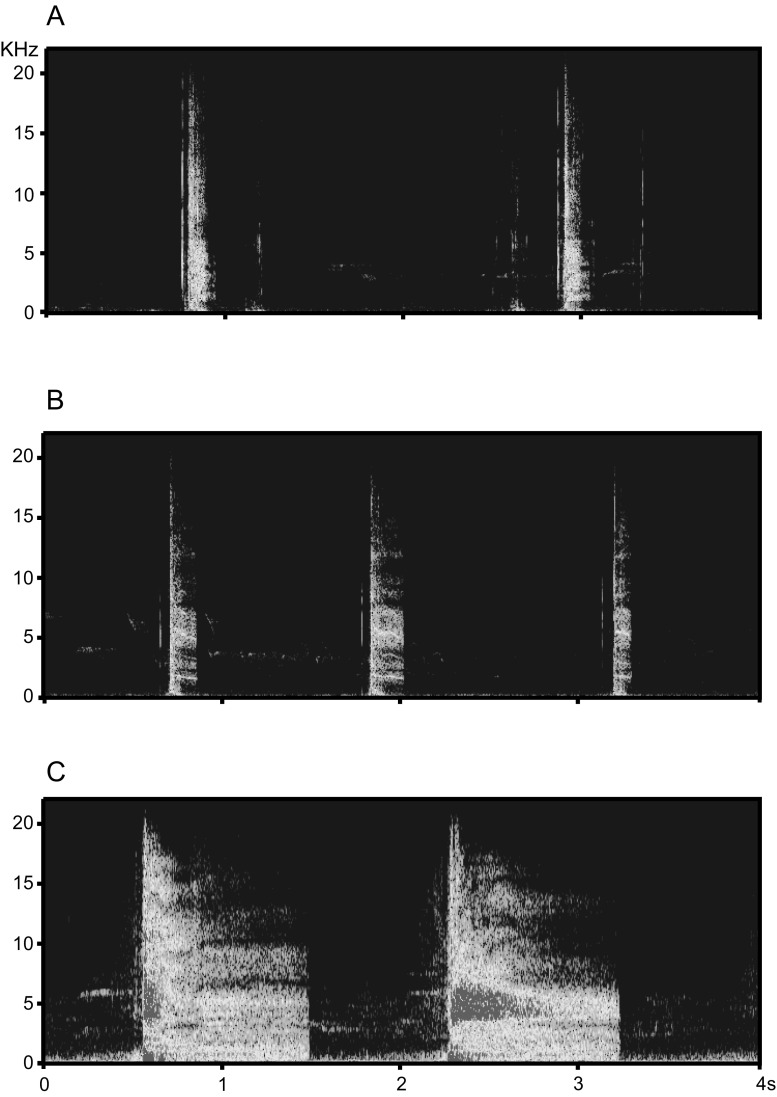
Sonograms (FFT length 512, sampling rate 44.1 kHz) of hissing calls made by Great Tit (**a**), Blue Tit (**b**), Marsh Tit (**c**), generated in SasLab Pro Avisoft, Germany

The hissing sounds of tits result from two simultaneous actions: shaking the wings and producing vocalisations. Since we were not able to distinguish the individual effects of these actions in the sound production, we treated them as an overall sound produced by the tits. Calls of Great Tits are short and resemble clicks while those of Blue Tits and Marsh Tits sound like a sharp exhale or hiss. Calls of each of the three species begin with short, broad-band clicks (Fig. 2). Calls of Great Tits do not contain any constant frequency elements while those of Blue Tits contain nearly solely constant frequency elements at 1.9, 3.7, 5.4, and 7.0 KHz (Fig. 2b). Calls of Marsh Tits consist of faint frequency-modulated elements (Fig. 2a). Call duration of Great Tits was shorter than of Blue Tits and Marsh Tits (Table 1). The time between successive calls was longer for Great Tits than for Blue and Marsh Tits (Table 1).

**Table 1:** Physical parameters of hissing calls of Great Tits (*N* = 3), Blue Tits (*N* = 3), and Marsh Tits (*N* = 3)

Measurements	Great Tit	Blue Tit	Marsh Tit
	mean ± SD	mean ± SD	mean ± SD
Duration [s]	0.18 ± 0.03	0.50 ± 0.28	0.83 ± 0.09
Time between calls [s]	1.97 ± 0.89	1.06 ± 0.42	0.95 ± 0.18
Peak frequency [kHz]	4.44 ± 1.38	4.51 ± 1.77	4.06 ± 0.19
Maximum frequency [kHz]	10.97 ± 2.61	17.69 ± 1.57	12.76 ± 1.22
Call rate (calls/min)	29	38	33

The peak frequency of calls was similar for all species, only slightly higher for Blue Tits and lower for Great and Marsh Tits (Table 1). The highest maximum frequency characterised the calls of Blue Tits, and the lower those of Marsh and Great Tits (Table 1). Call rate differed among species largely because of species-specific differences in the duration of pauses between consecutive parts of call. These pauses lasted about 1 s in Blue Tits and Marsh Tits and 2 s in Great Tits (Table 1, Fig. 2). The relative amplitude of calls was the highest for Great Tits, lower by 2.5 dB for Marsh Tits and by 9.6 dB for Blue Tits.

Experiments were performed in the evening, between 7 and 10 pm. All trials were recorded with a digital camera (Sony HDR-SR12, with own infrared light source) that was placed in front of the experimental arena. An independent source of infrared light was focused on the arena to allow us to video record the behaviour of mice in almost complete darkness. The duration of each trial was 10 min, and we noted the sex and age of each experimental animal. We analysed videos to determine the amount of time each mouse spent exploring the terrarium, the number of visits made to each cavity, and the amount of time spent in the experimental (with playback) and control cavities (without playback).

It was not possible to carry out observer-blind tests, because the study involved wild animals and an experienced person was needed to handle them. Also, videos were not scored by a “blind” observer, because data were collected over extended period of time (4 years) and we decided that the video data should be analysed only by one of the authors (KZ), as a means of minimising the variability that would be introduced by using multiple observers. Moreover, the parameters we measured (time durations and number of visits to cavities) are less susceptible to observer biased error than other types of measured behaviours. Consequently, we do not think that this approach significantly affected results of our experiment.

### Ethical statement

The mice were captured in Białowieża National Park during four periods: June 2012 (9 individuals), June 2013 (24 individuals), April 2014 (8 individuals), and June 2015 (31 individuals), using wooden live traps. Traps were set at dusk and captured mice were transported to the Mammal Research Institute the following morning, where they were individually housed in standard cages. The experiment was performed on the day after capture. In total, we captured 64 animals (36 females and 28 males, 39 adults and 25 juveniles), which were released at the site of capture after completing all procedures.

Yellow-necked mice were trapped using wooden box traps, baited with oats and carrot. Because this species is exclusively active at night, traps were opened in the evening and checked the next morning. Most trapping was performed in June, when nights were warm. In April, we ceased trapping when temperatures fell below 10 °C, to avoid mortality of animals due to cold. Mice were transported to the lab in the traps and placed individually in standard cages: dimensions ca 40 cm × 20 cm × 30 cm (W × D × H). We provided bedding (wood shavings), paper tubes to enrich environment, as well as food (grain, sunflower seeds, nuts) and water ad libitum. During the experiment, we placed the mouse in an open trap in the arena and waited until the animal left the box. The trap was immediately removed and the mouse freely explored the experimental chamber. When the experiment was completed, we again placed the trap in the arena and slowly directed the mouse into the trap to avoid direct contact with the animals and thereby minimise their stress. We performed experiments during the evening and released the mice the next morning at the place of capture. To prevent animals from being re-captured and tested multiple times, each mouse was marked with a small notch in the ear before release.

### Statistical analyses

Dependent variables in our analyses were the time spent by mice in a cavity with or without playback and the number of visits to cavities with or without hissing sound. Thus, number of samples equalled number of visits in cavities (*N* = 593 visits, 285 visits—cavities with sound, 308 visits—cavities without sound). We evaluated the significance of independent variables using a mixed model, where the full model included four fixed factors: presence or absence of playback (coded as a two-level factor), tit species (three species), age of mouse (coded as a two-level factor—juveniles or adults), sex of mouse, and four random factors: variant of tit call (three different records for each species), side of trunk location (coded as a two-level factor—left or right), animal ID, and the year of study. We modelled the effect of independent variables on number of visits to cavities. We used a Poisson distribution for these count data.

To test the effect of different calls of tits on the behaviour of mice, we ran a separate model using data for experimental cavities (with playback) only. We used a backwards stepwise approach and retained only significant factors (*p* < 0.05) in the final model. To test the significance of each factor, we used likelihood ratio tests. All continuous variables were log-transformed prior to analyses. Statistical analyses were carried out using lme4 package (Bates et al. 2015).

## Results

**Fig. 3:**
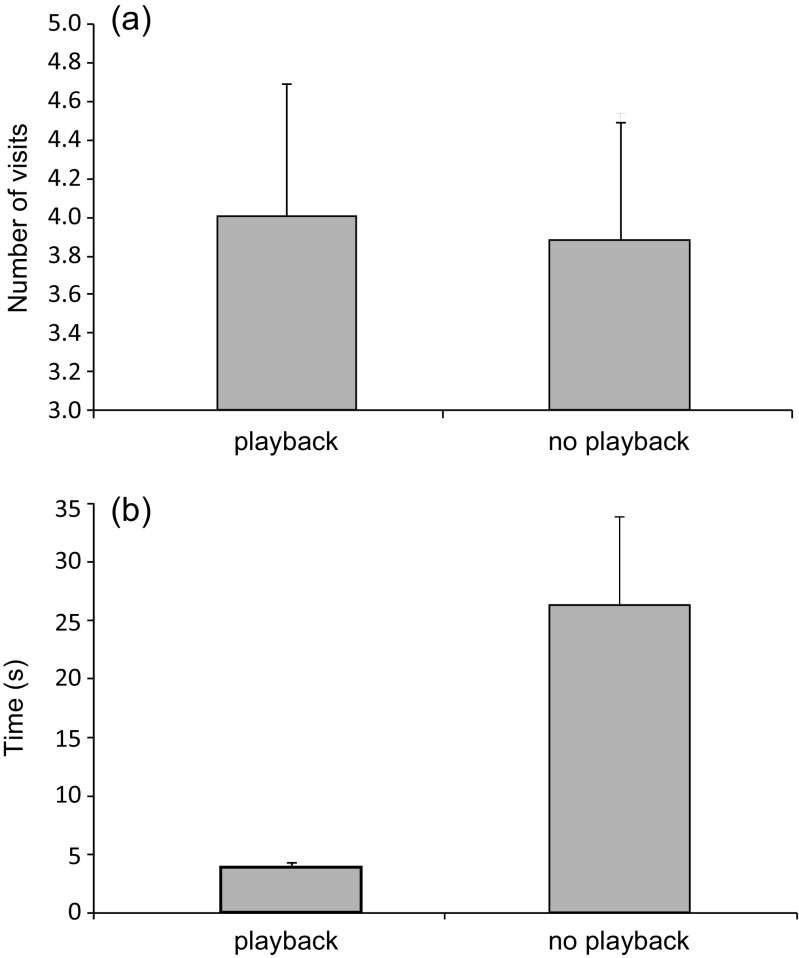
Mean number of visits (**a**) and mean time (**b**) spent by mice in cavities with playback of hissing calls (*N* = 285 visits) and without playback of hissing calls (*N* = 308 visits). *Bars* indicate 95% confidence intervals

During the 10-min trials, mice spent most of their time walking around the terrarium (on average of 6.86 min, SD = 3.32). Number of visits to the two cavities (with or without hissing sound) did not differ significantly. For cavities with hissing playback, the number of visits averaged 4.0 (SE = 0.34; 95% CI 3.3–4.7), whereas for cavities without playback visits averaged 3.9 (SE = 0.31; 95% CI 3.3–4.5; likelihood ratio = 0.0001, *df* = 1, *p* = 0.99; Fig. 3a). However, the time spent by mice in cavities with hissing playback vs. without was significantly different. Mice spent less time (mean = 3.91 s, SE = 0.21; 95% CI 3.50–4.32) in cavities with playback compared to without playback (mean = 26.34 s, SE = 3.87; 95% CI 18.73–33.95; likelihood ratio = 270.68, *df* = 1, *p* < 0.001; Fig. 3b). The remaining factors (bird species, sex, and age of mice) had no significant effect on the amount of time spent by mice in a cavity (likelihood ratio = −4.66, *df* = 2, *p* = 0.097; likelihood ratio = −3.13, *df* = 1, *p* = 0.077; likelihood ratio = −3.16, *df* = 1, *p* = 0.075; respectively) or on the number of visits (likelihood ratio = 0.88, *df* = 2, *p* = 0.644; likelihood ratio = 0.001, *df* = 1, *p* = 0.978; likelihood ratio = 0.76, *df* = 1, *p* = 0.384; respectively).

**Fig. 4:**
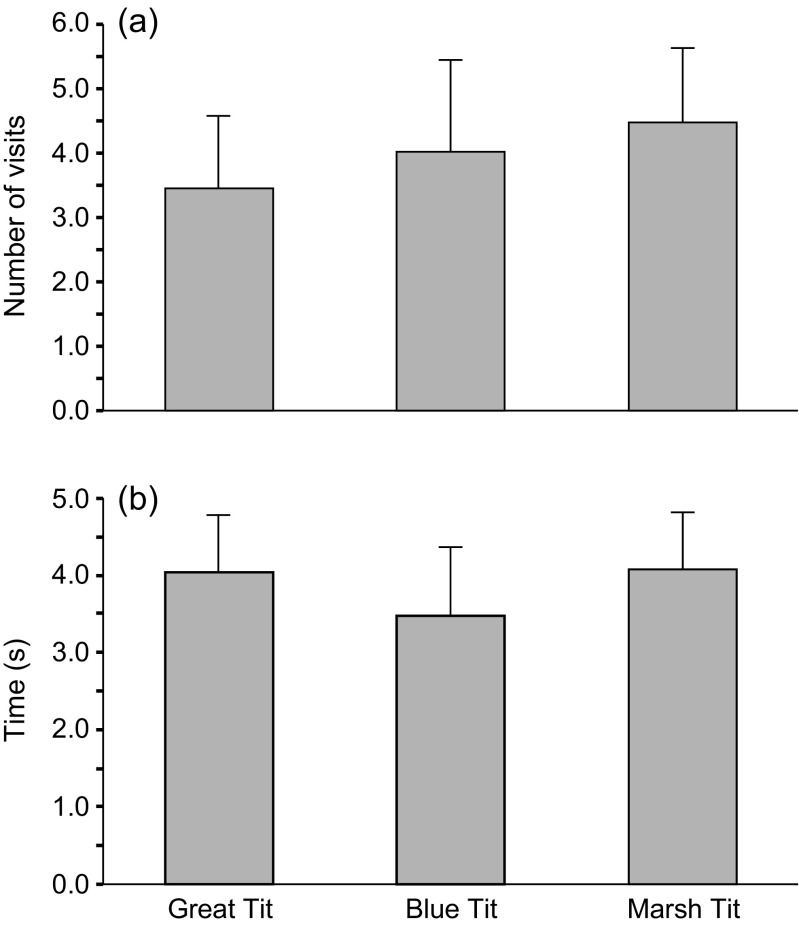
Mean number of visits (**a**) and mean time (**b**) spent by mice in holes with calls of different tit species (Great Tit, Blue Tit, and Marsh Tit). *Bars* indicate 95% confidence intervals

Analyses restricted to experimental cavities only (with hissing playback) revealed that on average mice spent a shorter amount of time in cavities with Blue Tit playback (3.49 s; SE = 0.44; 95% CI 2.60–4.38, *N* = 79) compared to in cavities with the Marsh Tit playback (4.09 s; SE = 0.38; 95% CI 3.34–4.84, *N* = 108) or in cavities with the Great Tit playback (4.05 s; SE = 0.38; 95% CI 3.29–4.81, *N* = 98). These differences were not significant (likelihood ratio = 7.09, *df* = 3, *p* = 0.07). Mean number of visits by mice in cavities with playbacks of different tit species varied from a mean of 3.5 to 4.5, but these differences were not significant (likelihood ratio = 0.49, *df* = 2, *p* = 0.783; Fig. 4). Again, we did not find a significant effect of the sex or age of a mouse on the amount of time spent by mice in the cavities (likelihood ratio = −3.66, *df* = 1, *p* = 0.056; likelihood ratio = −3.53, *df* = 1, *p* = 0.060; respectively) or the number of visits to cavities (likelihood ratio = 0.01, *df* = 1, *p* = 0.915; likelihood ratio = 0.45, *df* = 1, *p* = 0.504; respectively).

## Discussion

Our experiment revealed that the hissing calls of small birds, such as tits, can modify the behaviour of intruders resulting in shorter visits to a cavity. We suppose that hissing calls encourage the Yellow-necked Mouse to withdraw from the cavity and the shorter visits by mice to cavities with playback, compared to those without sound, suggested that the hissing of tits caused a rapid reaction by the mice to this sudden, intense stimulus.

The anti-predator properties of the hissing calls of tits seem to mimic the sounds made by a weasel or snake, which smaller nest predators like mice may mistake for potential danger (Sibley 1955; Apel and Weise 1986). Our experiment, however, revealed that mice returned to cavities with a hissing sound and even when revisiting these cavities, they spent significantly less time there than they do in those cavities without playback. Such a persistent reaction to previously encountered hissing calls may be especially important for the survival of mice given their high predation risk from larger, tree climbing predaceous mammals such as Pine Martens and Weasels (Zalewski et al. 1995; Walankiewicz 2002a). In Central Europe, there are no snake species that visit tree cavities, so it is very unlikely that the reaction of mice is related to the avoidance of snakes. However, we cannot exclude the possibility that hissing behaviour evolved very early in the history of this group of birds, when they were still exposed to predation by snakes. Consequently, additional experiments are needed to determine whether the response of mice to avian hissing calls can also be triggered by the hissing sounds of snakes, the squeaking sounds of weasels, or by any sudden sound.

According to Odum (1942), nestling Black-capped Chickadees *Poecile atricapillus* begin hissing at about 12 days of age, a few days before leaving the nest. This means that the startle strategy is adopted when juveniles are able to escape from the cavity, but they stay silent when younger and unable to flee from predators. Shortly before fledging, Great Tit nestlings respond differently to the alarm calls given by parents indicating different types of predators. Suzuki (2011, 2015) found that shortly before fledging, Great Tit nestlings responded differently to the alarm calls given for a snake (responded by jumping out of the nest cavity) and a crow (responded by crouching down inside the cavity). Frequent calls produced by nestlings can increase the chance of nest detection (Haff and Magrath 2011) Therefore, even if nestling tits were able to make hissing calls at earlier stages of life, they could decrease their probability of survival by disclosing their hiding place when they are too young to successfully escape the nest.

The most common nest-defence behaviour of female tits from inside the nest, which may be universal in the Paridae family, is the hissing display to nest intruders, but also visual displays and calls against potential predators entering cavities given from outside of the nest chamber (Broughton 2005, 2012). The results of our experiment demonstrate that hissing calls might be an effective anti-predator mechanism, but they raise the question of why this behaviour evolved only in some groups of species and not others.

Our experiment demonstrated how the hissing behaviour of Parids could potentially improve the breeding success of three common species by reducing the time that Yellow-necked Mice stay in a cavity. Other research corroborates our findings. Krams et al. (2014) showed that hissing female Great Tits had higher survival rates than silent ones and in the Białowieża Forest, the Collared Flycatcher *Ficedula albicollis* and Pied Flycatcher *F. hypoleuca*, which never hiss in the nest, usually have much higher breeding losses than tits (Walankiewicz 2002a; Wesołowski 2002; Czeszczewik 2004; Wesołowski and Rowiński 2012; Maziarz et al. 2016). Our results suggest that the behaviour of tits can change the exploration activity of the Yellow-necked Mouse, which may increase the breeding success of tits relative to the silent flycatchers. However, whether our findings apply generally to other nest predator species remains to be tested.
